# A Time Bomb in the Arm: Rare Delayed Presentation of a Post-traumatic True Brachial Artery Aneurysm

**DOI:** 10.7759/cureus.86102

**Published:** 2025-06-15

**Authors:** Preethika Murugesan, Jesu Pencilin Yesuvadiyan, Karthikeyan Selvaraj, Sasikumar Pattabi

**Affiliations:** 1 Medicine and Surgery, Mahatma Gandhi Medical College and Research Institute, Puducherry, IND; 2 General Surgery, Sree Balaji Medical College and Hospital, Chennai, IND; 3 Surgery, Sree Balaji Medical College and Hospital, Bharath Institute of Higher Education and Research, Chennai, IND

**Keywords:** arterial wall pathology, ct angiography, delayed vascular complication, end-to-end anastomosis, peripheral artery aneurysm, post-traumatic aneurysm, true brachial artery aneurysm, ulnar artery aneurysm, upper extremity aneurysm, upper extremity vascular injury

## Abstract

True aneurysms of the upper extremity arteries are rare, particularly in the brachial and ulnar arteries. We present the case of a 52-year-old male with a painful, progressively enlarging swelling over the left antecubital fossa, ultimately diagnosed as a true brachial artery aneurysm. Surgical excision and end-to-end arterial anastomosis were performed successfully. Histopathology confirmed the diagnosis of a true aneurysm. Incidentally, imaging also revealed an asymptomatic true aneurysm of the contralateral ulnar artery, for which conservative surveillance was advised. This case highlights the importance of considering vascular etiologies in upper limb swellings and underscores the potential for bilateral or multiple aneurysms in atypical locations. It also emphasizes the need for thorough systemic evaluation and long-term follow-up in such patients.

## Introduction

Aneurysms are defined as permanent localized dilations of an artery, typically measuring 50% greater than the normal vessel diameter. While peripheral artery aneurysms are uncommon, brachial artery aneurysms are particularly rare, constituting less than 1% of all peripheral aneurysms [1]. The brachial artery, the principal vessel supplying the forearm and hand, runs superficially and is susceptible to trauma. Nevertheless, true aneurysm formation characterized by involvement of all three arterial wall layers (intima, media, and adventitia) is exceedingly rare in this location, especially in the absence of systemic vascular pathology [2]. True aneurysms are generally associated with atherosclerosis, connective tissue disorders, infections, or repetitive trauma [3]. Conversely, pseudoaneurysms are more commonly seen post-trauma or after invasive procedures, resulting from partial vessel wall disruption and blood containment by surrounding tissue or adventitia [4]. True traumatic brachial artery aneurysms are rarely documented, particularly those following blunt injury. This case report presents a rare instance of a post-traumatic true brachial artery aneurysm with delayed presentation, along with an incidental finding of a contralateral ulnar artery aneurysm. We discuss the clinical presentation, diagnostic evaluation, and surgical management of this uncommon pathology.

## Case presentation

A 52-year-old male presented with a progressively enlarging, painful swelling over the left antecubital region. Two months earlier, he sustained a blunt trauma to the left forearm due to a fall from a two-wheeler, which was managed conservatively without vascular assessment. Fifteen days prior to presentation, the patient noted a dull aching pain localized to the swelling, particularly exacerbated by elbow movement. He had no known comorbidities.

**Figure 1:**
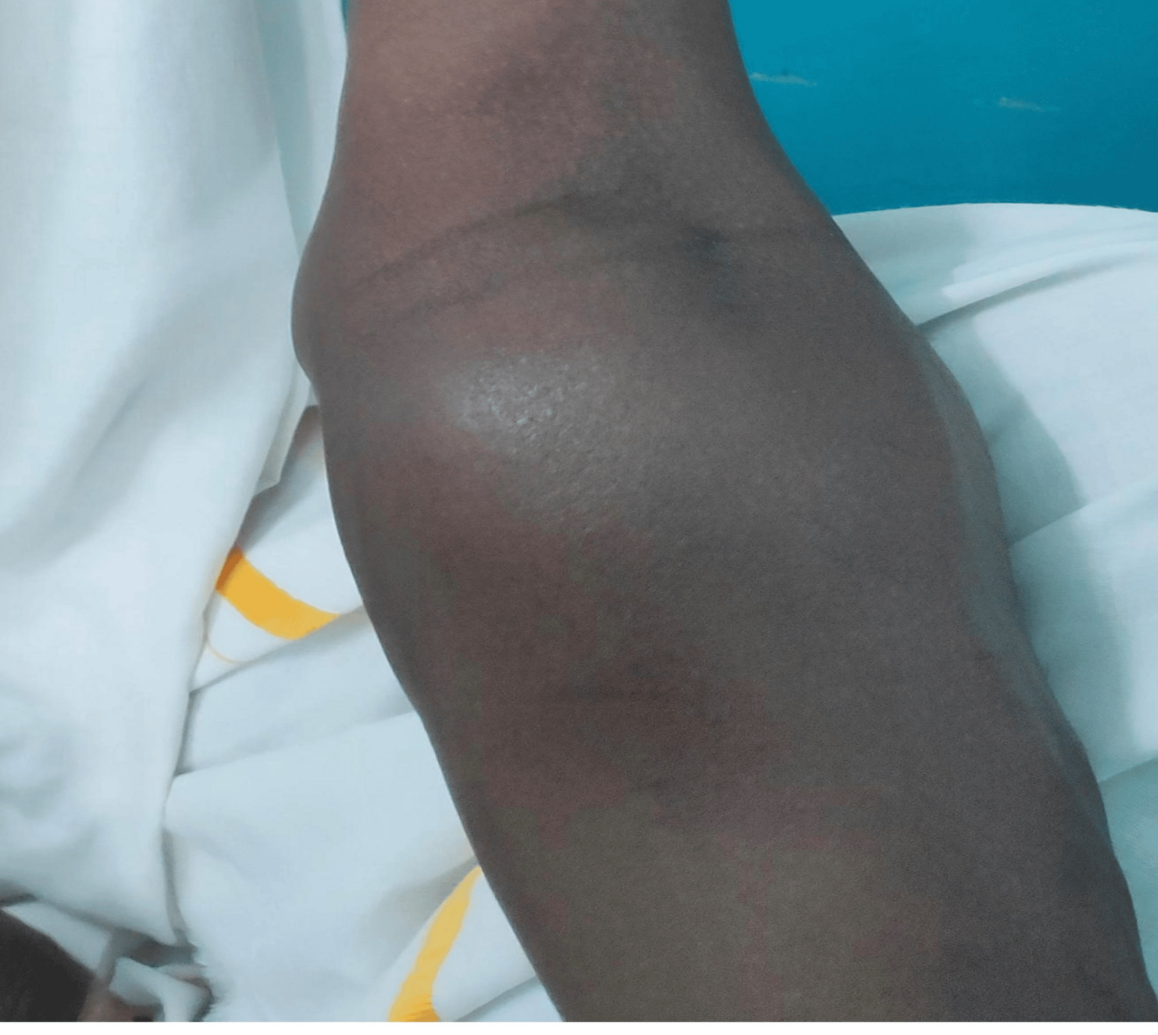
Clinical image showing superficial swelling over the left antecubital fossa

On examination, vitals were stable; systemic findings were within normal limits. A 6x5 cm soft, pulsatile, and compressible swelling was noted on the anteromedial aspect of the proximal third of the left forearm (Figure 1). Mild erythema was present over the swelling; a palpable thrill and continuous bruit were noted. Distal radial and ulnar pulses were symmetric and well-perfused. Capillary refill was normal; Allen’s test was positive, indicating adequate collateral supply. No sensory or motor deficits were observed; tone and reflexes were intact bilaterally.

Investigations

**Figure 2:**
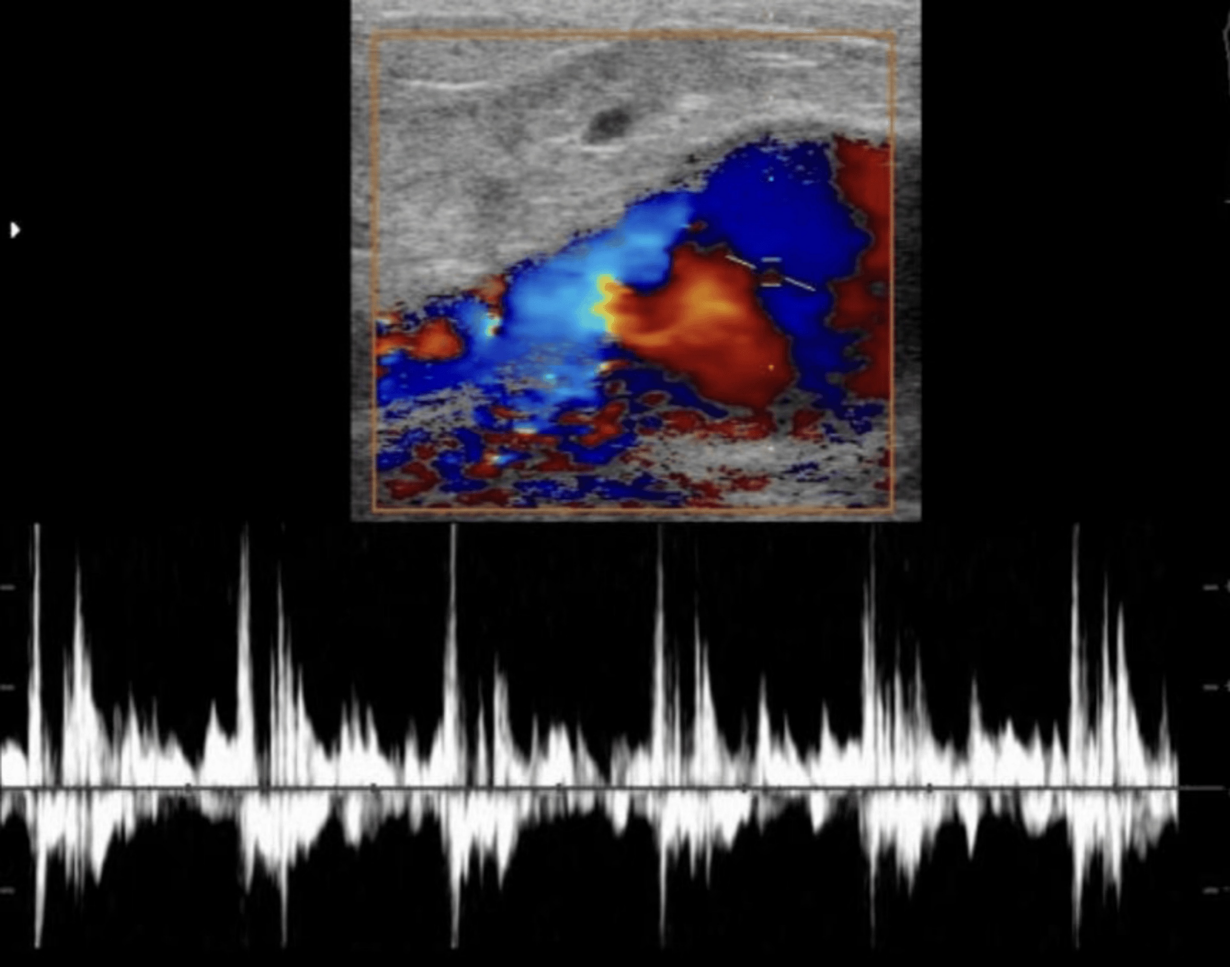
USG duplex showing the brachial artery aneurysm with turbulent flow

**Figure 3:**
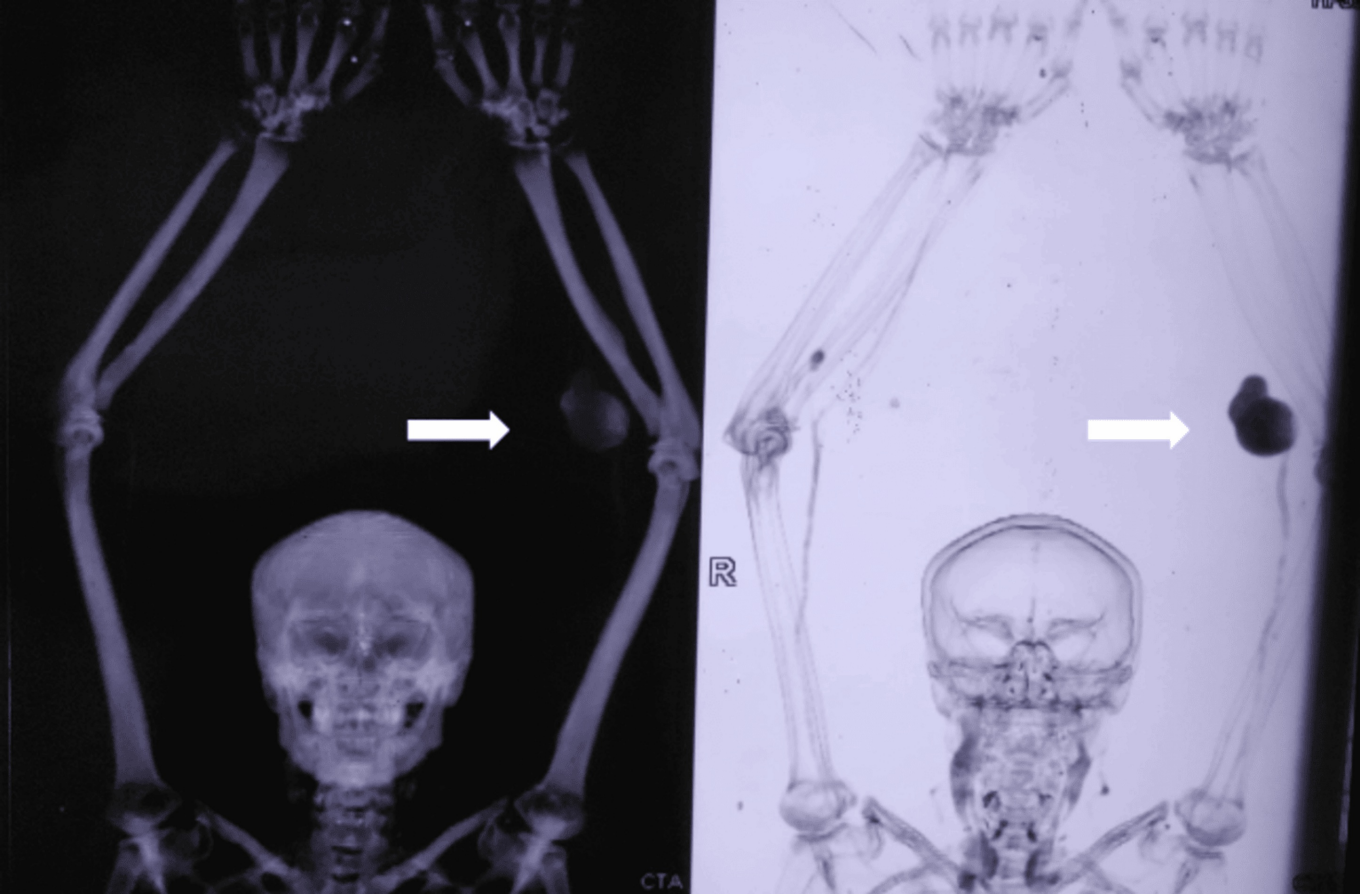
CT angiogram highlighting the B/L upper limb peripheral artery aneurysm

**Figure 4:**
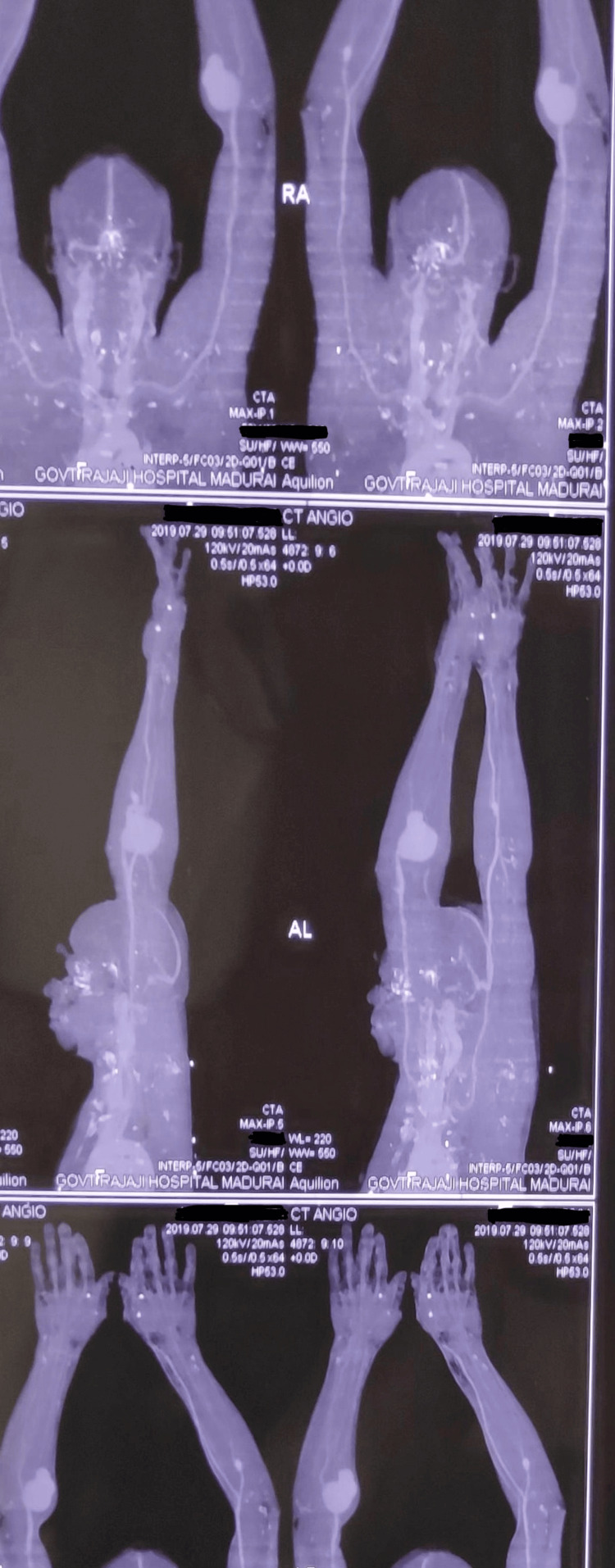
CT angiogram of the upper limb showing the left brachial artery aneurysm and right ulnar artery aneurysm

A Duplex ultrasound revealed a saccular aneurysmal dilatation of the left brachial artery with turbulent flow (Figure 2). A CT angiogram confirmed a 6x4 cm aneurysm proximal to the bifurcation of the brachial artery, with no thrombus or distal embolization. Incidentally, a 2x2 cm saccular aneurysm was detected in the right ulnar artery, just distal to its origin. Based on these findings, a diagnosis of symptomatic true brachial artery aneurysm was made, and the patient was scheduled for surgical intervention (Figures 3-4).

Intraoperative findings

**Figure 5:**
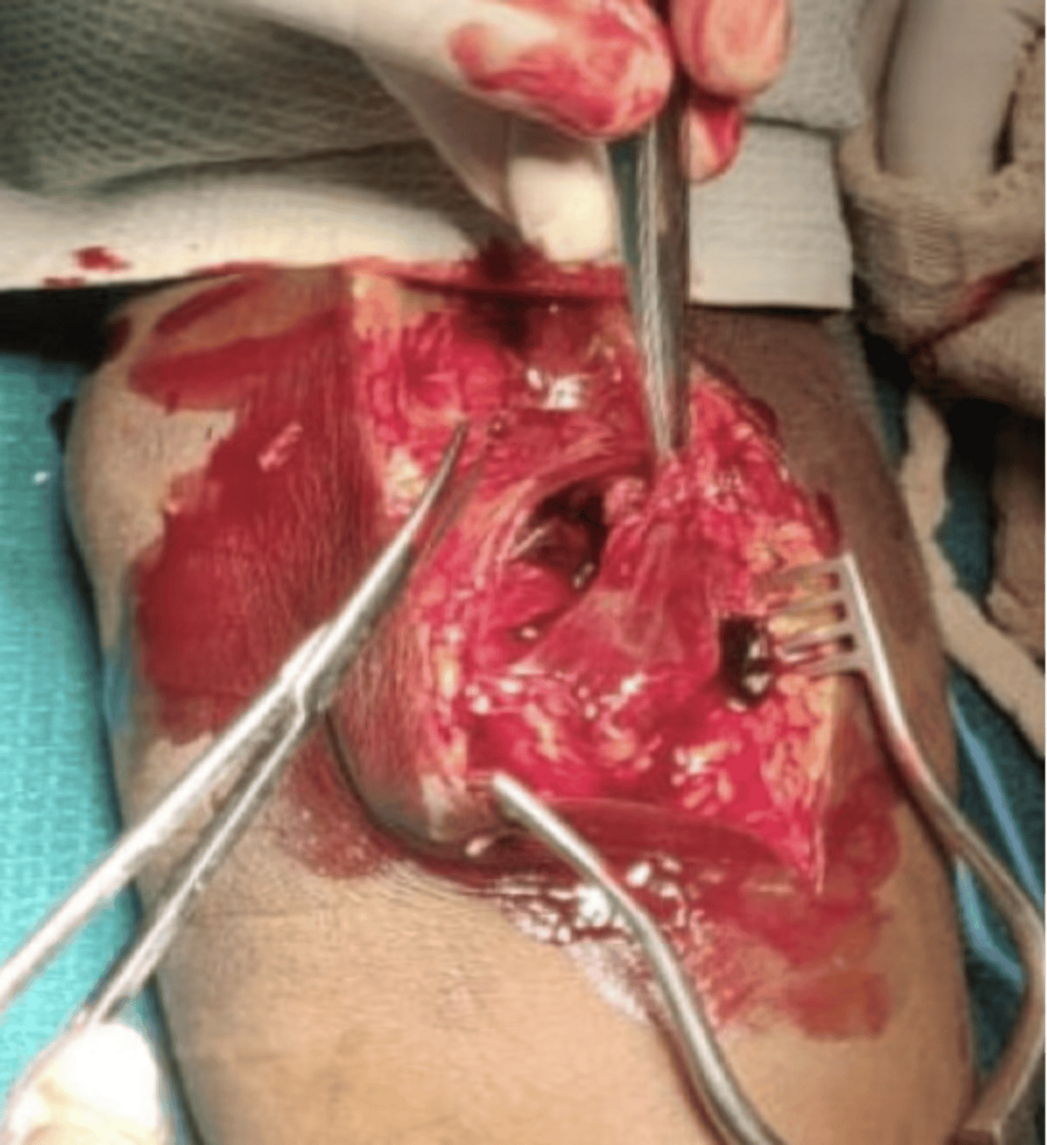
Intraoperative resection of the brachial artery aneurysm

**Figure 6:**
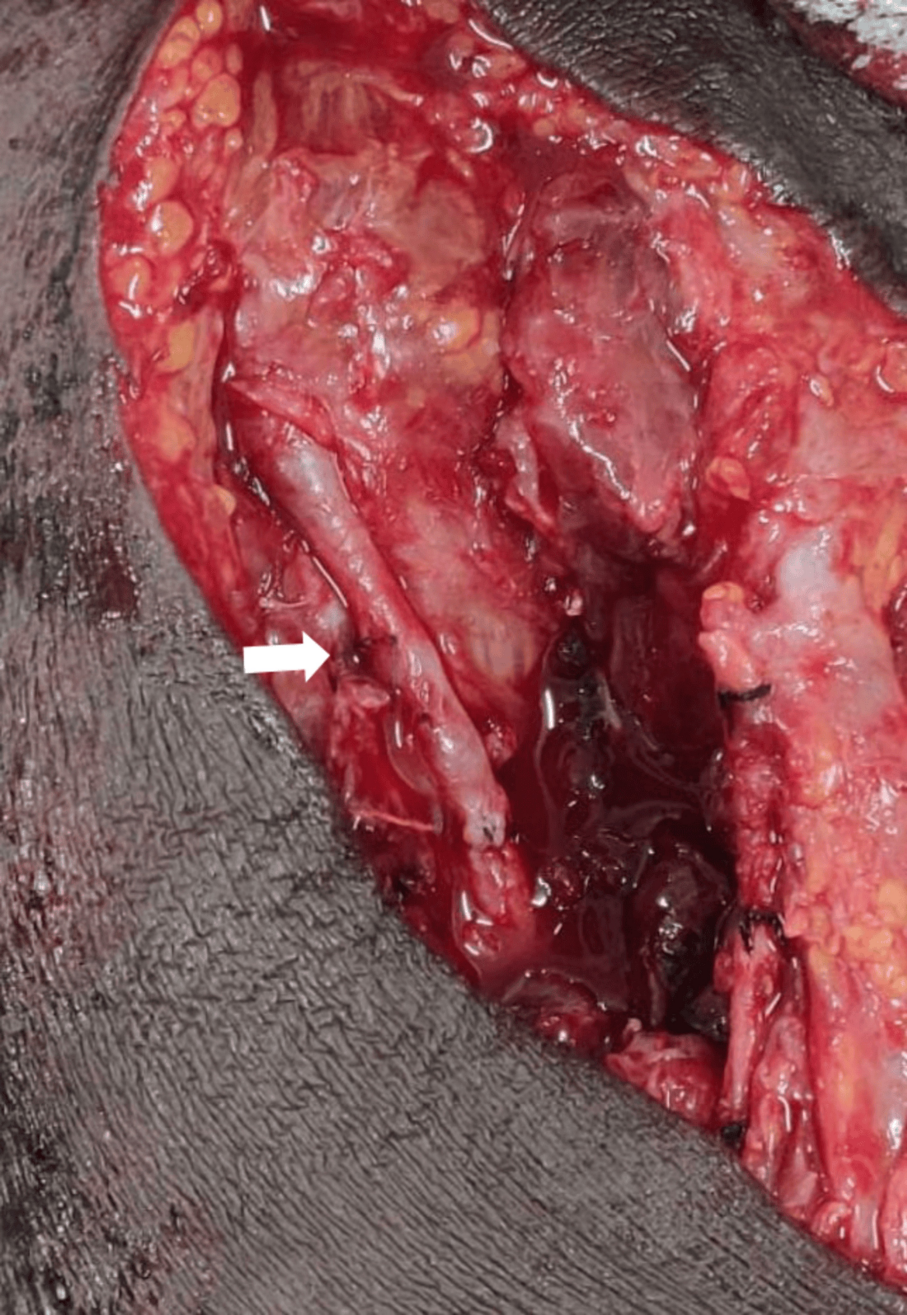
Intraoperative end-to-end anastomosis of the brachial artery

Surgical exploration confirmed a 6x4x3 cm aneurysm proximal to the bifurcation of the brachial artery (Figure 5). The proximal and distal segments of the artery were healthy and without thrombus. After vessel control, the aneurysmal segment was resected, and end-to-end anastomosis was performed using native vessel ends (Figure 6). The procedure was uneventful.

Postoperative findings

**Figure 7:**
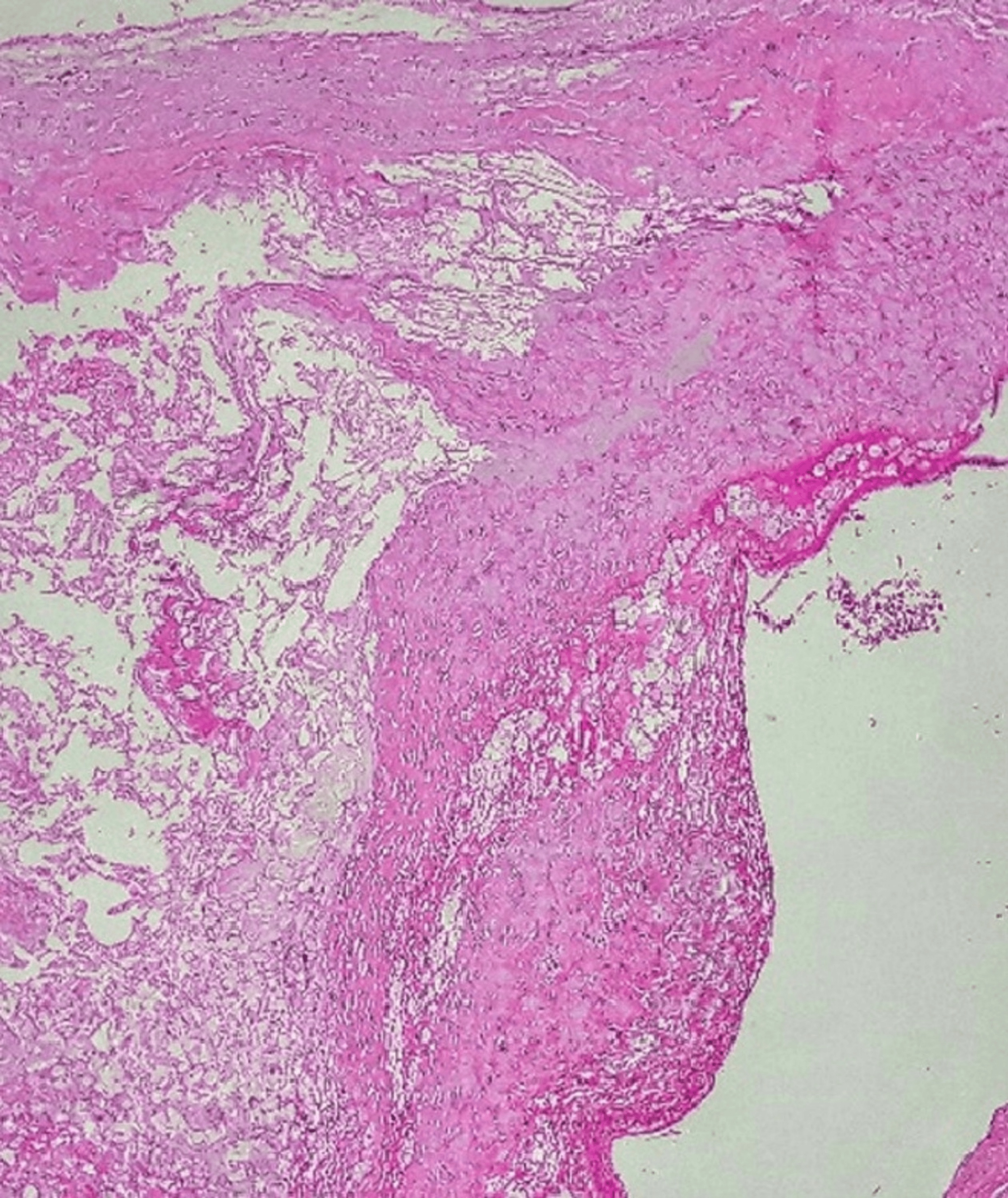
HPE showing features suggestive of true aneurysm HPE: Histopathological examination

Histopathology shows a section stained with hematoxylin and eosin with low-to-medium magnification (approximately 10x-20x objective) of the excised aneurysm, demonstrating all three arterial wall layers, with the tunica media replaced by hyalinized connective tissue, consistent with a true aneurysm. Adventitial thickening was evident, likely due to a reparative response to trauma (Figure 7). Microbiological cultures were negative for infection.

Postoperative Doppler ultrasonography revealed normal triphasic flow in the reconstructed left brachial artery. The radial and ulnar arteries showed biphasic flow. The contralateral ulnar artery aneurysm remained stable at 2×1.3 cm, with no flow compromise.

## Discussion

True aneurysms of the brachial artery are extremely rare, particularly those of traumatic origin [1,2]. While the brachial artery is a relatively superficial vessel and therefore prone to injury, the development of a true aneurysm - defined as a focal arterial dilation involving all three layers of the vessel wall: intima, media, and adventitia - following blunt trauma, is an uncommon occurrence [3]. In contrast, pseudoaneurysms (false aneurysms), which involve a breach in the arterial wall with blood collection confined by the surrounding soft tissues or adventitia, are more frequently reported following trauma or iatrogenic interventions [4,5].

This case illustrates a rare delayed presentation of a post-traumatic true aneurysm, with an additional incidental finding of a contralateral ulnar artery aneurysm - underscoring the complexity and diagnostic challenge of such vascular presentations. The delayed onset of symptoms in our patient suggests a gradual degenerative process of the arterial wall rather than an acute disruption. Trauma-induced true aneurysm formation typically follows intimal injury, which may initiate a cascade of medial necrosis and adventitial remodeling, eventually compromising the structural integrity of the vessel wall [6]. In some cases, these changes remain clinically silent until the aneurysm reaches a significant size, causing mass effect or pain due to local compression [7]. The blunt trauma sustained by the patient likely initiated this pathophysiologic process, ultimately resulting in aneurysmal dilation over a period of weeks.

Histopathological examination in this case revealed replacement of the tunica media by hyalinized connective tissue, confirming a true aneurysm. Additionally, cellular proliferation in the adventitia and lack of infectious growth on culture support a chronic reparative response rather than an acute infectious or inflammatory etiology [8].

The diagnosis of brachial artery aneurysms, especially true ones, can be challenging due to their rarity and subtle early symptoms [3]. The differential diagnosis of a swelling in the antecubital fossa includes soft tissue tumors, abscesses, ganglions, lipomas, hematomas, and pseudoaneurysms, each with distinct clinical and imaging features [9]. A pulsatile mass with an audible bruit, as in this case, strongly suggests a vascular etiology. Duplex ultrasonography serves as an excellent initial imaging modality, identifying the vascular nature of the lesion and assessing flow characteristics [10]. In this patient, duplex imaging demonstrated turbulent flow within a saccular aneurysm, prompting further evaluation. CT angiography remains the gold standard for definitive diagnosis and preoperative planning, offering detailed visualization of the aneurysm morphology, vessel integrity, and distal run-off [10].

Of particular interest was the incidental identification of a right ulnar artery aneurysm, which, although asymptomatic, raises several important clinical considerations. The presence of multiple arterial aneurysms, even in the absence of systemic vascular disease, warrants further investigation for underlying connective tissue disorders, vasculitis, or genetic predispositions [11]. Although the patient had no personal or family history suggestive of such conditions, these findings underscore the need for comprehensive vascular imaging and possible rheumatologic or genetic consultation in similar cases.

The management of brachial artery aneurysms depends on several factors, including symptomatology, size, risk of complications (e.g., rupture, thrombosis, distal embolization), and anatomical location [12]. According to vascular surgical guidelines and literature reviews, surgical intervention is generally recommended for symptomatic aneurysms, aneurysms >2 cm in diameter, evidence of thrombus or distal embolization, and progressive enlargement on surveillance [12,13].

In this case, the aneurysm was symptomatic, enlarging, and sizable (>6 cm), thereby meeting the criteria for surgical excision. Intraoperatively, the aneurysm was amenable to primary end-to-end anastomosis due to healthy proximal and distal vessel segments. This is considered ideal when tension-free anastomosis is feasible, as it eliminates graft-related complications and offers good long-term patency [13]. Alternative surgical options include autologous vein grafting (usually the great saphenous vein), synthetic grafts (e.g., polytetrafluoroethylene (PTFE) or Dacron) in cases where the vein is unsuitable or unavailable, and bypass procedures for complex or multifocal aneurysmal disease [14].

Postoperative recovery was uneventful, and follow-up Doppler studies demonstrated restored triphasic flow in the reconstructed brachial artery. The radial and ulnar arteries showed biphasic flow, likely due to postoperative hemodynamic adaptation.

The discovery of a contralateral ulnar artery aneurysm, although asymptomatic, introduces a diagnostic dilemma. While it may represent a coincidental finding, the possibility of pre-existing subclinical arterial wall vulnerability must be considered. One hypothesis is that hemodynamic redistribution following injury to one limb may result in compensatory stress on the contralateral arterial tree, predisposing to aneurysm formation [15]. Another plausible explanation is the presence of a genetic predisposition such as fibromuscular dysplasia, Marfan syndrome, Ehlers-Danlos syndrome, or segmental arterial mediolysis, even in the absence of overt clinical signs [16]. While no evidence of systemic disease was found in this patient, continued surveillance of the contralateral lesion and consideration of systemic screening are prudent. Management of the asymptomatic ulnar aneurysm was conservative, with periodic imaging and symptom monitoring advised. Intervention would be indicated if the lesion becomes symptomatic, enlarges significantly, or demonstrates thrombotic changes [14].

A comprehensive literature review reveals that most published cases of brachial artery aneurysms are pseudoaneurysms resulting from penetrating trauma, catheterization, AV fistulas, or surgical procedures [4,5]. True aneurysms, particularly of traumatic origin, are seldom reported [2,3]. In a 21-year review by Zelenock et al.,brachial artery aneurysms accounted for less than 1% of peripheral arterial aneurysms, and most were associated with chronic dialysis or systemic diseases [1]. Only a minority were true aneurysms secondary to blunt trauma. A case similar to ours was described by Sultan et al., who reported the delayed presentation of a traumatic brachial artery aneurysm in a young adult [7]. However, our case is unique in presenting with bilateral upper limb aneurysms, one symptomatic and one incidental, both likely related to vascular injury or degeneration.

This case serves as a reminder of the need for long-term vascular follow-up in patients who sustain significant limb trauma, even in the absence of initial vascular findings. The subtle evolution of true aneurysms over time can be missed if not actively monitored. Additionally, clinicians should maintain a high index of suspicion for vascular injuries in patients presenting with late-onset limb swellings, particularly when accompanied by pulsatility or bruit. Finally, this case emphasizes the importance of detailed imaging of the entire vascular system in any patient diagnosed with a peripheral artery aneurysm, given the potential for silent contralateral or multifocal involvement [11,14].

## Conclusions

This case underscores the rarity and clinical complexity of true brachial artery aneurysms, particularly those developing insidiously after blunt trauma. The delayed onset and subtle symptomatology present diagnostic challenges that demand high clinical suspicion. The concurrent incidental finding of a contralateral ulnar artery aneurysm raises important considerations regarding possible underlying arteriopathies or compensatory hemodynamic changes. Early vascular imaging and comprehensive assessment are vital in any patient presenting with persistent or unexplained upper limb swellings, especially those with a history of trauma. Duplex ultrasound and CT angiography play pivotal roles in prompt diagnosis and surgical planning. Surgical resection with primary end-to-end anastomosis remains the preferred treatment in anatomically favorable cases, offering excellent outcomes. Finally, this report emphasizes the necessity of long-term vascular surveillance in patients with peripheral aneurysms, including asymptomatic contralateral lesions, to prevent catastrophic complications such as rupture or distal ischemia. A multidisciplinary approach incorporating vascular surgery, radiology, and, when indicated, genetic or rheumatologic evaluation is essential for optimal management and follow-up.
